# Rheology and Microstructural Behavior of Semidilute Suspensions of Semiflexible Rods across Five Decades of Shear Rate

**DOI:** 10.1021/acs.macromol.4c02675

**Published:** 2025-02-18

**Authors:** Paul F. Salipante, Steve Kuei, Ryan P. Murphy, Jeffrey A. Fagan, Christopher M. Sims, Katie M. Weigandt, Steven D. Hudson

**Affiliations:** † Polymers and Complex Fluids Group, Materials Science and Engineering Division, National Institute of Standards and Technology, 100 Bureau Dr, Gaithersburg, Maryland 20899, United States; ‡ National Center for Neutron Research, National Institute of Standards and Technology, 100 Bureau Dr, Gaithersburg, Maryland 20899, United States

## Abstract

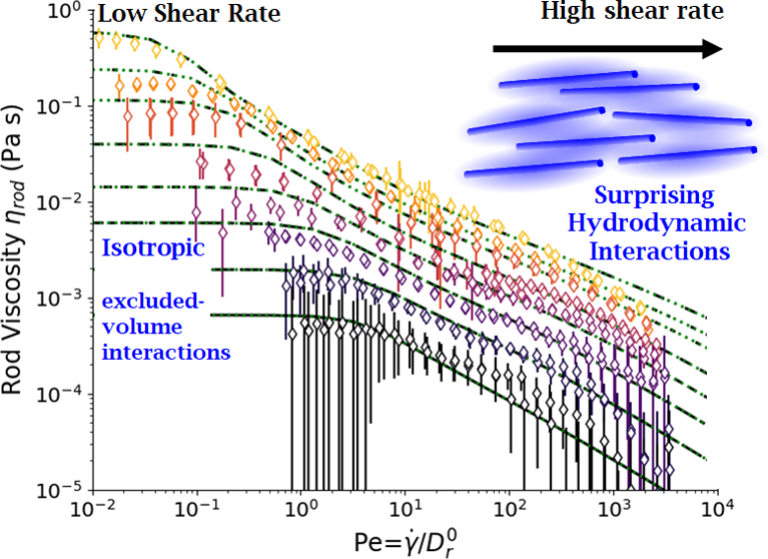

Rod-like particles are efficient rheology modifiers and are commonly found in a variety of biological and industrially relevant suspensions, from biofilaments to worm-like surfactant micelles. These suspensions display strong shear-thinning behavior, and ongoing efforts aim to understand the microstructural changes of these fluids: how they depend upon the properties of the suspended particles and how these changes manifest in the resulting rheology. With suspensions of fd bacteriophage as a model monodisperse rod system, we use capillary microrheometry and flow birefringence to determine rheological behavior across nearly six decades of shear rate up to 6 × 10^5^ s^–1^, at various semidilute concentrations. A single, and surprisingly large, primary fitting parameter accounting for the characteristic distance of hydrodynamic interactions is consistent with viscosity data up to very high shear rates. These results may prompt other work to understand the mechanics of these interactions.

## Introduction

The flow behavior and stability of complex fluids in shear are commonly studied; this is particularly true of suspensions of long filaments due to their ability to efficiently viscosify. Strong shear thinning, and in some cases flow instabilities, have been observed in filamentous suspensions, for example, of rigid filaments such as actin,^1^ semiflexible polymers,^2,3^ and materials with transitory fibrous and sometimes branched structures.^4^ There are ongoing efforts to understand how microstructure drives bulk flow dynamics.

In the simplest abstractions to theoretically describe their rheological behavior, these filaments can be treated as either coiled (when flexible) or rod-like (when stiff). The focus of this work is rod-like filaments. At rest, rod-like fluids have clear equilibrium phase transitions as a function of rod concentration.^5^ However, when subjected to flow, the rod orientations are affected, which leads to shifts to higher concentrations in the dilute, semidilute, and concentrated transition regimes and introduces the possibility of a mechanical origin of transition.^6−10^ These microstructural changes produce strong non-Newtonian rheological behavior and can potentially generate dynamical instabilities, such as shear bands.^11^

Theoretical approaches begin with Jeffery’s calculations for the equations of motion of dilute ellipsoids in shear flow.^12^ In higher concentration suspensions, two realms of interparticle interactions, excluded volume and hydrodynamics, have been explored. The direct excluded-volume interactions limit the diffusive motion and decrease the effective diffusivity. Calculating the effects of these two types of interactions has mainly been along two separate lines of research. Furthermore, it has been argued that hydrodynamic interactions may be neglected when the rods are long and thin.^13^ It will nevertheless be necessary in this report to use both of these interactions together.

Concerning colloidal Brownian suspensions,^7,8^ the Fokker–Planck equation describes the competition between shear and rotational diffusion coefficient through an orientational distribution function (ODF). As rod concentration is increased to reach the semidilute regime, however, neighboring rods can hinder each other’s rotational freedom, leading to the need to include a pair-correlation function to properly describe the relationships between the structure and dynamics. Lang et al.^14^ compared two promising theoretical calculations for rigid-rod fluids with measurements from rheology with small-angle neutron scattering (rheoSANS). Assuming a pair-correlation function that is independent of shear rate, Lang et al.^14^ use this ODF to derive a relationship between the microscopic orientational ordering tensor and macroscopic measurables such as the zero-shear viscosity η_0_ and the onset of shear thinning. Another theory, by Doi–Edwards–Kuzuu (DEK),^7,15^ uses a phenomenological function to add orientation dependence to the rotational diffusion coefficient, and the most promising recent theories extrapolate to infinite shear on the assumption that rods align enough to behave as if they are dilute. The ODF can also be obtained from scattering measurements by rheoSANS based on an orientation-dependent particle scattering model.^16^ The moments determined from the ODFs were then used in a bulk stress calculation and compared to those in bulk rheology. Lang et al. measured the shear viscosity of fd-virus suspensions at shear rates up to approximately 100 s^–1^.^17^ The shear thinning behavior they observed suggested that the tube model, which describes direct interparticle interactions in semidilute conditions, is not applicable at high alignment because the rods can rotate more freely.

While this understanding (and neglect of hydrodynamic interactions) works well for shear rates up to 100 s^–1^ or 1000 s^–1^, here we test these ideas further by exploring very high shear rates, up to approximately 1 × 10^5^ s^–1^. We achieve these rates using capillary rheometry and flow birefringence capabilities that were recently developed.^18−21^ By doing so, we are able to explore these shear-induced “dilution” effects experimentally and investigate the effects of hydrodynamic interaction. We compare this new data with theoretical models to help establish the more complex outlines of a full understanding of the rheology of a semidilute suspension of rods. Interaction between rods will be discussed and remains surprisingly significant here, suggesting that rotations remain hindered.

We choose the fd-virus as a quasi-ideal model system. It has a length *L* = 880 nm, diameter *d* = 6.6 nm, and a persistence length *l*_*p*_ = 2.8 μm.^14,22,23^ Since the aspect ratio is *L*/*d* = 133, and the persistence length is roughly 3 times the length, the fd-virus is a remarkably thin, stiff, and monodisperse rod, making for an excellent and well-studied model.^16,23−27^ In addition, both the effective aspect ratio^28^ and the flexibility^22^ of the virus can be tuned, allowing for future exploration.

The rest of this paper is structured as follows. First, we will summarize the theories describing the rheology of rod-like fluids, in particular, how semidilute flow behavior may be calculated from the orientational distribution of the suspended particles, how dilute and infinite shear behavior may be calculated from the Fokker–Planck equation for the orientational distribution, and how various theories describe hydrodynamic interactions between rods. Second, we demonstrate the ability of capillary rheology and flow-induced birefringence to test fluids at high shear rates.

## Theory

**Figure 1:**
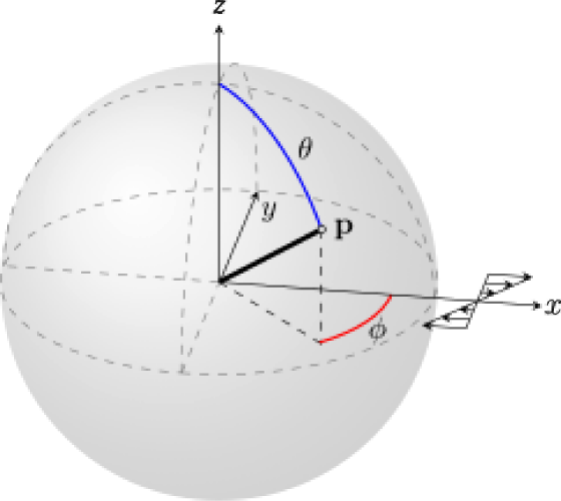
Polar angles θ and ϕ, describing the orientation vector **p** of a given rod. Shear flow is shown with the flow in the **x** direction, velocity gradient in the **y** direction, and vorticity axis in the **z** direction.

The response of a Brownian suspension of monodisperse rigid rods to an applied shear flow was originally developed by Doi and Edwards^29^ and Hess.^30^ Similar work for spheroidal particles was developed by Hinch and Leal.^31,32^ These methods use a modified Fokker–Planck equation to predict the rod orientation in the direction **p**, making an angle θ and ϕ with the flow coordinates, as seen in Figure 1, from its probability density function *N*(θ,ϕ), which is the probability of finding a rod with an orientation in the orientation (θ,ϕ).

In order to describe the change in *N*(θ,ϕ) when flow is applied, Hinch and Leal described the evolution at a given shear rate γ̇ as^31,32^
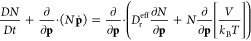
1

 is the effective rotational diffusivity, *k*_B_*T* is the thermal energy, *V* is an interaction potential, and **ṗ** is the evolution of **p** in flow over time:
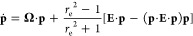
2where **E** is the rate of strain tensor, **Ω** is the vorticity tensor, and *r*_e_ is the effective aspect ratio of the rod. While this aspect ratio is derived from spheroidal particles, the effective aspect ratio for a cylindrical rod has been calculated to be^33^
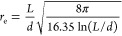
3where *L* is the rod length and *d* is its diameter.

The free rotational diffusivity for a rod from slender-body theory is
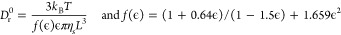
4where η_s_ = 1.218 mPa s is the viscosity of D_2_O solvent at 21 °C and ϵ = [ln(2*L*/*d*)]^−1^. A value of  = 16.5 s^–1^ is calculated for the specified dimensions of the fd in D_2_O. With these dimensions, ϵ = 0.18; *f*(ϵ)=1.58.

Equation 1 can be solved numerically and can be rearranged into an evolution equation for the second moment, defined as
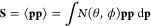
5

For reference, we compute the evolution of **p** numerically (eq 2) using the finite-volume method in the dilute limit, where no interactions between particles are considered.^34^ The orientations of the rod are discretized from −π/2 < (ϕ, θ) < π/2 on a 200 by 200 grid, and the solution is integrated forward in time using the Crank–Nicolson method until a steady-state distribution is reached.

The interactions between rods must be averaged in such a description as shown in eq 5. Following from Doi, a two-particle mean-field Maier–Saupe potential is^35,36^
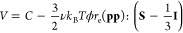
6where ν is an empirical constant of order one and *C* is a constant. By including this interaction potential into eq 1, an evolution equation can be derived for the orientation tensor:
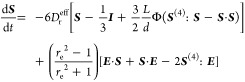
7and the fourth moment is defined **S**^(4)^ = ⟨**pppp**⟩, *E* is the strain rate γ̇/2, and Φ is the volume fraction of particles.

The direct interactions between rods at equilibrium also reduce the diffusive motion. Doi^35^ derived an expression for the effective rotational diffusion coefficient that scales as , where *n* is the number density. Under flow, the effective diffusion coefficient can increase as interactions between rods become less frequent with increasing alignment. An effective diffusion coefficient was proposed by Lang et al.:^17^
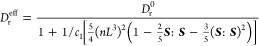
8where *c*_1_ is the interaction parameter that has been calculated to be approximately 1.35 × 10^3^, from a mean-field Green-function formulation.^37^ The effective diffusion coefficient increases as the alignment increases. Other methods developed by Witteman have related the contact points between rods, which are related to the eigenvalues of the orientation tensor.^38^ The differences between these approaches result in minor differences in the onset of an increase in diffusivity with rod alignment.

The remaining component of eq 7 to address is the fourth moment, ***S***^(4)^. An evolution equation for the fourth moment would include higher moments, and to therefore close the expression, the term is typically approximated in terms of combinations of the second moment. Significant efforts have been made to produce equations that accurately approximate the fourth moment at low and intermediate shear rates.^36^ There has been less attention to the accuracy at high shear rates, in particular the representation of the second Newtonian plateau expected at very high Peclet number, .^39,40^ For fd, this Pe limit is expected above *Pe* > 4 × 10^5^. Other closure approximations overestimate the magnitude of the second Newtonian plateau and result in a reentrant Newtonian behavior at intermediate *Pe* numbers, e.g., the Dhont–Briels closure.^13,36^ The hybrid closure provides a nonfitted closure approximation for the fourth moment without this behavior because it transitions from a linear approximation at low shear rates to a quadratic approximation at high shear rates:
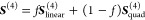
9where  is the Hand closure,  = ***SS*** is the quadratic form, and the function *f* = 27 det(***S***) varies from 0 for isotropic configuration to 1 when aligned. The hybrid closure approximation is chosen for this work because it more accurately describes the dominant response of the quadratic term in the range of Peclet numbers, particularly around *Pe* ∼ 10^3^. While it does not predict a second Newtonian plateau, the range of *Pe* numbers accessible in experiments is below 10^4^. The second Newtonian plateau predicted by Hinch and Leal (1972) depends on the rod aspect ratio. For the fd system with aspect ratio *r*_e_ ≈ 74, this prediction gives an intrinsic viscosity of approximately [η]_inf_ = 10.

The stress for an axisymmetric particle can be derived from the disturbance of the flow caused by the particle. The particle stress is described by the second and fourth moments.^33^ For long rods, the equation for stress can be simplified to three main terms. Two contributions arise from the deformed structure, under shear, of Brownian diffusion and excluded volume interactions, described by an effective potential between rods, which adds to the stress. The other contribution is the average particle stresslet term, which describes the hydrodynamic stress of rods under flow. The coefficient for this stresslet term has been derived for different concentration limits, particle shapes, and orientations.^13,29,39,41^ Hydrodynamic interactions are typically neglected due to the relatively small contribution to the stress expected from hydrodynamic interactions compared with other stresses. These three contributions result in the following equation, written in terms of number density *n*,^13^
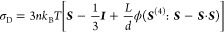
10
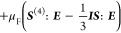
10bwhere μ_F_ is a factor that describes the effective contribution to the viscosity from the rod stresslet. For Brownian suspensions, this term is typically taken from the dilute hydrodynamic description. While this assumption may be valid up to an intermediate *Pe* number, at a high *Pe* number hydrodynamic interactions may become significant as the stresslet becomes large compared to the other terms. When hydrodynamic interactions between rods are taken into account, this term can change depending on the spacing and orientation of the rods. Following the terminology from Mackaplow and Shaqfeh,^42^
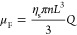
11where *Q* is a term that varies depending on the distance between particles and will be used here as a fitting parameter that we will compare to various predictions. For example, for the dilute assumption, a cylindrical rod has
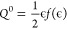
12which, with fd’s dimensions, here equals 0.14. The dilute regime can be related to the free diffusion coefficient by
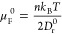
13

The transition from dilute to semidilute would be expected from excluded volume interactions to be at *nL*^3^ = 1, but the slender shape allows the rods to more easily avoid each other and experimentally the semidilute limit becomes significant at *nL*^3^ ≳ 30.^43,44^ Hydrodynamic interactions also between particles become significant at similar concentrations. An *O*(*nL*^3^) correction to the dilute theory was developed by Mackaplow and Shaqfeh to account for two-body interactions,^42^
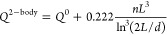
14which may be significantly larger than *Q*^0^.

Batchelor proposed a general description of the hydrodynamic interaction between rod particles as a function of an interparticle screening length χ:^45^
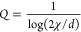
15where χ depends on the number density, rod length, and the orientation. Several calculations were then proposed to determine this screening length. If the characteristic distance between rods is taken as the closest approach, the average shortest distance to a neighboring rod, then this distance depends significantly on alignment. For aligned rods, the distance is inversely related to , but the scaling becomes different for the isotropic case.^7^ The scaling depends on the excluded volume but can be written in a general equation as,
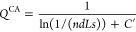
16where *s* is an adjustable length; *s* = *L* when the orientation is isotropic and *s* = *d* when the system is aligned, and *C*’ is an *O*(1) constant, which may be positive or negative, that represents a higher-order term and can be calculated in more detail as a function of fiber shape and orientation. We use this equation here with *C*’ = 0 and adjust *s* as approximate bounds for comparison with results. Note that this equation is not expected to be accurate near its singularity, i.e., at the upper end of the semidilute concentration range. Shaqfeh and Frederickson predicted a value for *Q* based on the average interparticle distance (i.e., not closest approach) between rods in either an aligned or isotropic configuration using a multiple scattering analysis. They developed the expression in terms of volume fraction,
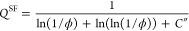
17where *C*” = 0.16 for aligned rods and *C*” = −0.66 for isotropic rods. Because alignment changes with the shear rate, we expect that the interaction length may change with the shear rate as well. As noted above, we use the value of *Q* as a fitting parameter and compare to the predictions using eqs 14, 16, and 17.

The total stress includes the solvent contribution, **σ**_T_ = 2η_s_***E*** + **σ**_D_. For a given shear rate, eq 10 allows us to readily calculate the stress and therefore the theoretical viscosity η = σ_T,*xy*_/γ̇ of a rod suspension. We will compare our results presented as the rod viscosity,
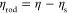
18

For semidilute rod suspensions, we expect that low shear behavior will be dominated by direct rod–rod interactions and have significantly higher viscosity than dilute predictions. However, with increasing shear rates, shear-induced alignment will lower each rods’ effective excluded volume, causing the suspension to approach dilute behavior at high shear.^17^ However, if the concentration is high enough and the alignment not complete, then shear-induced rod–rod collisions remain.^38,46^ We shall see indeed that the highly aligned high-shear rheology surprisingly suggests a substantial hydrodynamic interaction.

### Birefringence

The birefringence is measured at each imposed pressure through the center of the microcapillary along a path length of the diameter. Therefore, this measurement, in the 1–3 (*x*–*z*) plane, is similar to one across the gap in a plate–plate rheometer. In contrast to that measurement, however, this measurement in the capillary traverses a linear gradient in stress (i.e., through the Poiseuille profile), so that the measured retardance is an integral of the birefringence sampled across that linear function of stress. The mean birefringence is the total retardance divided by the capillary diameter. In this geometry, the following orientation is sampled:^47^
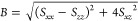
19

We note that in this parallel shear flow, the value *S*_*xz*_ is zero, and therefore the change in the birefringence due to orientation is related to (*S*_*xx*_ – *S*_*zz*_) only.

The value of *B* functions as an order parameter that describes the transition from an isotropic orientation (*B* = 0) to a fully aligned orientation (*B* = 1).^48−50^ The birefringence is proportional to the product of the order parameter and the saturation birefringence,
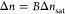
20

The saturation birefringence Δ*n*_sat_/*c* = 3.8 × 10^–5^ L g^–1^ was measured from the liquid crystal phase in which *B* is nearly unity.^51^ The measurements are compared to model predictions (eq 10) of *B* at shear stress values sampled on the linear gradient. To do so, from capillary rheometry measurements of the steady shear viscosity over a range of applied pressures, the total stress σ_T_ and flow yield the relationship between rod stress σ_D_ and shear rate.

## Materials and Methods

### Fluid Rheology

Viscosity was measured with a custom capillary rheometer.^18^ This small-volume capillary rheometer applies a known pressure drop to submilliliter volumes of sample via an Elveflow OB1 microfluidic flow controller^a^ across a length of capillary (Molex Polymicro Capillary Tubing, (100 and 200) μm diameter). The capillary flows into a larger glass capillary (*d* = 1.12 mm, WPI), allowing tracking of the liquid front using a line sensor (Hamamatsu). Converting the liquid front velocity to flow rate and assuming that the fluid resistance of the large capillary is negligible compared to that of the small-diameter flexible capillary, we can then calculate the viscosity of the fluid; further details can be found in Salipante et al.^18^ By applying pressure drops, Δ*p*, between 0.5 and 500 kPa, shear rates above 10^5^ s^–1^ can be achieved. At the highest shear rate of approximately 10^5^ s^–1^ and with the 200 μm diameter capillary, the highest estimated Reynolds number for a fluid with a viscosity of 1 mPa s is approximately 200, roughly an order of magnitude lower than the transition to turbulence in pipe flow.

### Flow Induced Birefringence

Flow birefringence measurements were performed using a Photron Crysta polarization-sensitive high-speed camera, whose superpixel size is 40 μm. Within each superpixel is a 2 × 2 array of pixels having individual polarizers arranged in 45° increments. Monochromatic light at wavelength λ = 525 nm from a Thorlabs DC2200 was directed through a Thorlabs 532 nm quarter-wave plate (WPQ20ME–532), the sample flow cell, a 2× objective (Leica 10447178 PLANAPO 2.0x/WD 39 mm), and a Leica Z16 APO into the camera, in the order given, for a total magnification of 46×. The scale in the image is therefore 0.87 μm/superpixel. We then calculate the retardance *R*, which describes the phase shift of the incoming circularly polarized light, with the background retardance (approximately 0.6 nm) of a quiescent sample subtracted out.

The sample flow cell is a round 100 μm diameter (*a*_c_) silica capillary length *l* = 70 mm. This capillary with fittings was mounted on a rigid aluminum frame. Since the outside surface of the capillary has a protective coating of polyimide, this coating was removed by burning it with a torch. In this way, the incident light could pass through pure silica and the sample fluid only. The image ROI was (512 × 17) superpixels (445 × 14.8 μm). Sample reservoirs were connected to the capillary through short (50 mm) tubing (ID = 0.8 mm). The pressure drop through these tubes is negligible.

The wall stress in the silica capillary is σ_w_ = Δ*pa*_c_/(4*l*), and the wall shear rate is , where the apparent wall shear rate is , and *V̇* is the flow rate.^52^ The apparent wall shear rate is the true wall shear rate if the fluid viscosity is constant with shear rate, i.e., the fluid is Newtonian. The wall shear rate is used to define the *Pe* number for the capillary rheology measurements .

For the light path through the center of the capillary, the stress profile is linear. The total pressure drop was adjusted from 200 Pa to 680 kPa. A pressure pulse sufficiently long to reach steady-state flow and alignment (typically several seconds and up to nearly one min) was applied, so that the steady-state retardance and its relaxation upon flow cessation were recorded.

### Materials

Wild-type fd bacteriophage was grown at the Biomaterials Facility at Brandeis University in 2xYT growth medium following standard biological protocols by inoculating *Escherichia coli*. The resulting phage was purified by ultracentrifugation, suspended in Tris D_2_O buffer, and adjusted to 100 mmol/L ionic strength by using NaCl. We note that samples are prepared in D_2_O buffer for separate scattering measurements on the same stocks. The molar mass of fd is approx (14.6 ± 0.6) × 10^6^ Da.^53^ The concentration of fd was measured by UV spectroscopy (Nanodrop), based on an extinction coefficient of 3.84 cm^2^/mg at 269 nm.^53^ Prior to UV measurement, the parent suspensions were diluted by a known factor, e.g., 10, so that the absorbance could be measured accurately. The concentration of the parent suspension was determined accordingly. These measurements were repeated by preparing three different dilutions of each suspension, the concentration of which was measured in this way. Suspensions were stored at 2 °C to 8 °C whenever not being studied, over a period of more than 3 years.

The particle size distribution, of pristine and sheared samples diluted in ananlogous Tris H_2_O buffer, was evaluated by analytical ultracentrifugation (AUC) in a Beckman Coulter XL-I instrument at 20.0 °C through sedimentation velocity experiments monitored by absorbance detection at 269 nm. Analysis was performed using the “c(***s***)” model in the software package SEDFIT (V16.1c).^54−57^ Values for the (Tris H_2_O) buffer solution density and viscosity were measured with an Anton Paar DMA 5000 M–Lovis 2000 ME densitometer–microviscometer at 20.0 °C as 1002.664 kg/m^3^ and 1.015 mPa s, respectively. The initial concentration was experimentally verified through a concentration series to effectively yield dilute-limit sedimentation coefficients and is approximately one-third the theoretical volume-filling concentration of a sphere scribed by a fully rotated fd rod. Note that the difference in our reported peak sedimentation coefficient (≈ 42 × 10^–13^ s) and that of Dogic et al. (≈ 45 × 10^–13^ s) is entirely attributable to the difference in temperature of the experiments, mainly through the temperature dependence of the solvent viscosity.

Images of fd particles were also obtained by atomic force microscopy (AFM). Silicon-wafer substrates were functionalized with (3-aminopropyl)triethoxysilane by vapor treatment^58^ to create a positively charged surface for negatively charged fd bacteriophage, which was then deposited onto these substrates from a dilute (≈ 0.001 g/L) sample. 50 μL of this dilute sample was placed on the treated substrate and allowed 3 min for adsorption. Excess sample was rinsed by a stream of buffer, and the substrate was dried with compressed nitrogen gas. Imaging was done with ScanAsyst probes scanning an 8 μm square at 0.5 Hz.

## Results and Discussion

### Particle Characterization

**Figure 2:**
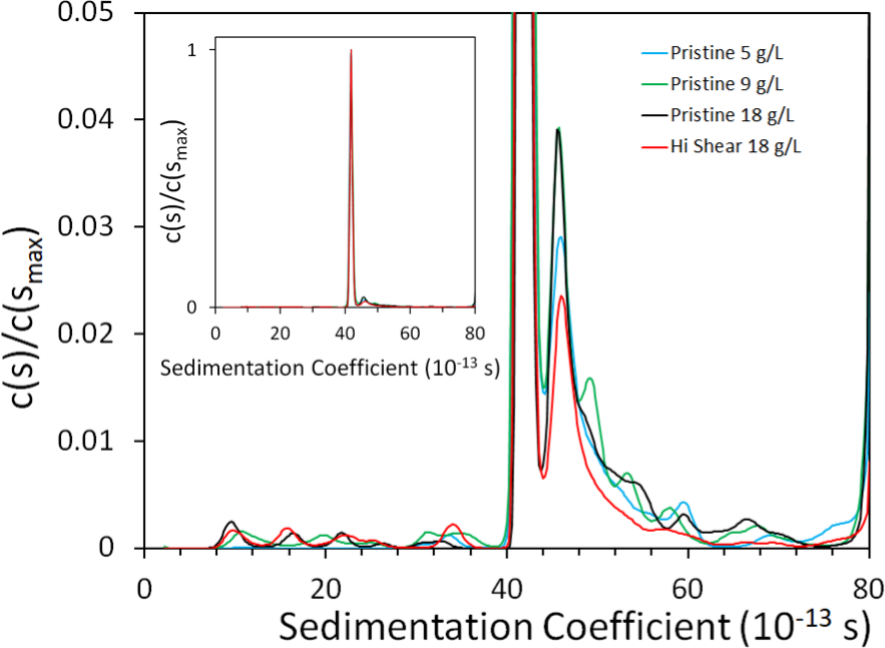
AUC measurements of the fd sample distributions. The inset shows the spectrum at full peak height and the main figure has expanded vertical scale to focus on the minor peaks.

We first characterize the shape and defect populations of the fd virus. The sedimentation coefficient profile measured by AUC comprises a single sharp peak (Figure 2 inset) corresponding to a rod approximately 900 nm long. The next most prominent feature (accounting for 9% to 15% of the total intensity in different samples) is one or more peaks from species that sediment approximately 10–20% faster than the primary peak. The identity of these species is uncertain; they may be defective rods, prone to bending, or they may be aggregates of two or three rods that have a similar ratio of mass to drag coefficient.^59^ It is conceivable that this population may be one source of the enhanced viscosity (to be reported in the following, Rheology section), greater than the prediction of isolated rods, when the shear rate exceeds ≈2000 s^–1^. The proportion of this population is essentially independent of the overall parent suspension concentration. Finally, in most samples, there is also a small amount of signal distributed into more slowly sedimenting components (accounting for 0.3% to 1.9% of the total intensity). These may be fragments of broken rods. In all cases, however, fd dispersions, whether highly sheared or pristine, are observed to be extremely similar and composed mainly of the isolated semiflexible rods, with only small differences in the minor impurities of similar hydrodynamic behavior or extremely small fractions of smaller rods. The equivalence of samples regardless of shear history suggests that conditions that might cause changes to fd particles are more extreme than what we have achieved here.

**Figure 3:**
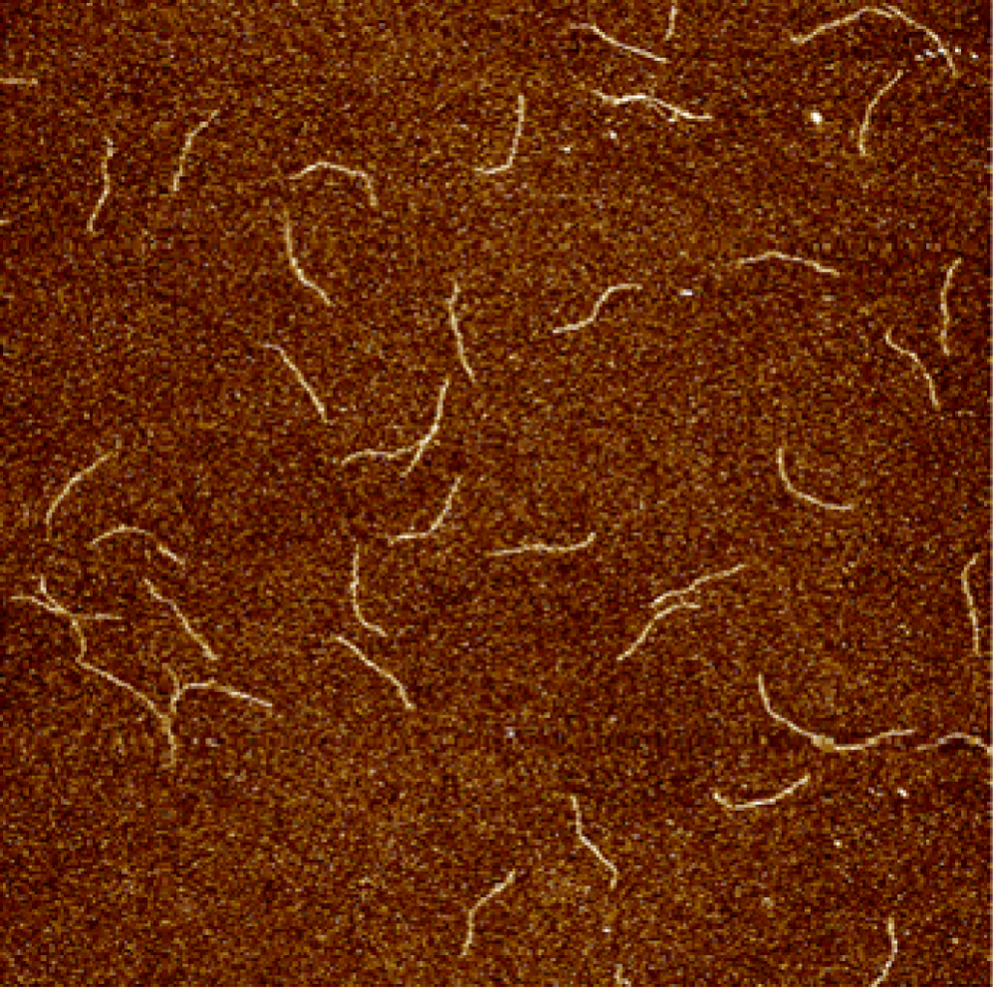
An 8 μm square AFM image of an fd sample taken from a sample that had been sheared up to 6 × 10^5^ s^–1^ at 9.0 g/L.

A sample image of the fd particles is shown in Figure 3. From multiple images, statistical analysis of 1701 nonoverlapping filaments finds a mass-average mean contour length of (784 ± 209) nm. This value is lower than expected. This difference may arise from the difficulty in clearly resolving the rods and, especially, their ends.

### Rheology

Several semidilute suspension concentrations are studied here; each concentration is above the overlap concentration, which is approximately 0.07 g/L. At this ionic strength, the isotropic phase is stable at rest up to approximately 21 g/L, beyond which it locally becomes spontaneously aligned in a cholesteric (chiral nematic) phase.^60^

**Figure 4:**
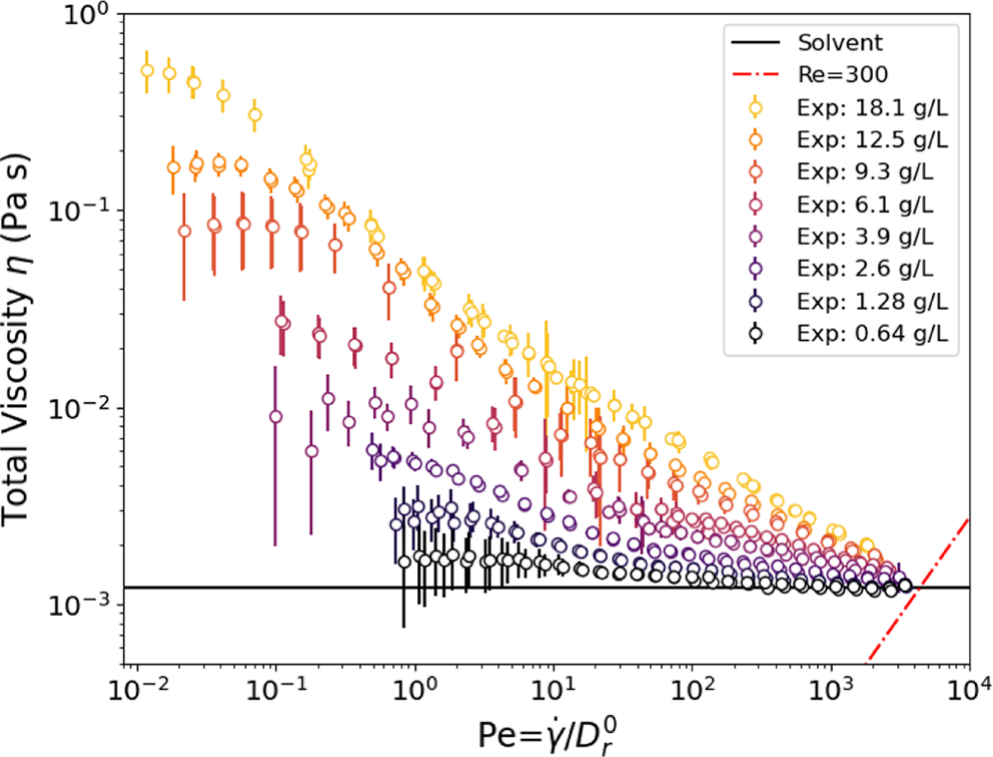
Total viscosity of concentrations of fd varying from 18.1 to 0.64 g/L for shear rates spanning nearly 6 decades, obtained by microcapillary rheometry. The black line indicates the D_2_O solvent viscosity. The minimum slope of log(stress) vs log(viscosity) is −0.7.

We begin with the viscosity measurements of the suspensions (Figure 4). Using capillary rheometry, we attain very high shear rates, over 10^5^ s^–1^, while also reaching a lower limit of approximately 1 s^–1^. Of particular note is that all samples continue shear thinning beyond the typical maximum shear rates of rotational rheometry, ≈10^3^ s^–1^, and approach a viscosity comparable to the solvent viscosity at the highest shear rates. The capillary rheometry measurements do not vary significantly with different-sized capillaries, indicating negligible entrance effects. These measurements also do not depend on shear history; results are the same regardless of the shear rate. To test whether flow-induced stress on the fd virus may result in their breakage, viscosity measurements were repeated, and no changes in viscosity were observed, regardless of sample history.

The magnitude of shear thinning increases with concentration, consistent with previous work by Lang et al.^61^ At higher concentrations, the suspensions shear thin somewhat strongly, and the steepest shear thinning occurs at shear rates of ≈10 s^–1^, i.e., roughly near *Pe* ≈ 1. We find that the highest concentration measured has a shear thinning coefficient ≈−0.7, which is not close enough to a value of −1 to exhibit shear banding.

**Figure 5:**
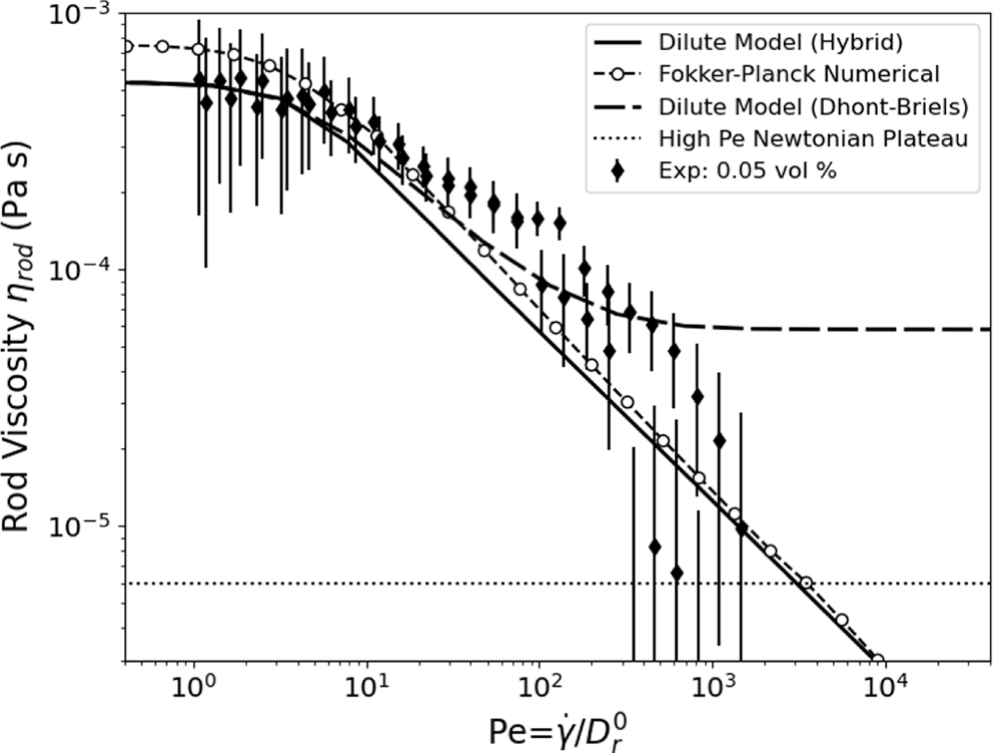
Rod viscosity as a function of *Pe* number for 0.64 g/L, 0.05 vol %, fd compared to theoretical predictions. The circle-dashed line indicates predictions from the numerical calculation of rod theory, and the solid line shows the viscosity based on the stress from eq 10. The dotted line shows the high *Pe* number Newtonian plateau predicted by Hinch and Leal (1972) for an effective aspect ratio of *r*_e_ = 74.^39^ The model with the Dhont–Briels closure is shown in a dashed line.^62^

We turn now to analyzing the data of Figure 4, compared to the dilute and semidilute models introduced previously in the Theory section. The experimental viscosity of the lowest concentration, 0.64 g/L, suspension (0.05 vol %) is shown in terms of rod viscosity (η_rod_) and compared with numerically calculated predictions for dilute rod suspensions (see Figure 5). The concentration may also be expressed in terms of the number density *n*, so that here *nL*^3^ ≈ 10 indicates that the rods in this suspension are weakly overlapping and at the transition to semidilute behavior. The dilute model assumption uses the free diffusion coefficient and viscosity coefficient of eq 12. The numerical Fokker–Planck simulation is for a single rod. In this dilute limit, shear-thinning arises from an increase in alignment, which in turn decreases the hydrodynamic disruption of the base shear flow and decreases the effective particle rotation from Brownian motion that contributes to stress at weak alignment. The agreement between the model and the numerical calculation at high shear rates validates the use of the model’s hybrid closure approximation. The model and simulation differ only slightly. The data show reasonably good agreement with the theory, particularly for the zero-shear-rate viscosity and the transition to shear thinning. The experimental data shows a slightly higher viscosity than the dilute model at high shear rates, but we also note the viscosity values are within 10% of the solvent viscosity and the measurement uncertainties become significant.^18^ This lowest concentration is at the transition to the dilute behavior. At lower concentrations, the rod contribution to viscosity is less than a few percent of the solvent viscosity and is thus not easily measured with our rheometer, as configured.

We have not achieved high enough shear rates to show the predicted second plateau of the rod viscosity,^40^ when the motion of the rod is essentially as defined by Jeffery.^12^ Since the aspect ratio of fd is large, the transition to this second Newtonian plateau is predicted to be at very high shear rates, specifically *Pe* ≈ 10^5^. The estimated magnitude of the viscosity on this second Newtonian plateau is shown as the dotted line in Figure 5, which for a volume fraction 0.05% is [η]ϕη_s_ ≈ 10^–5^ Pa s, less than 1% of the solvent viscosity.^39^ The theory shown in Figure 5 ignores the terms related to this plateau but intersects with it at *Pe* ≈ 10^4^. This is significantly lower than the high shear rate limit set by *Pe* > (*L*/*d*)^3^+(*L*/*d*)^−3^, though we note that an undershoot was observed in simulations of rods before reaching this Newtonian plateau at even higher shear rates.^40^ The high aspect ratio of the fd makes the system challenging for the exploration of this high shear plateau behavior. Furthermore, its semiflexibility is an additional challenge.

### Interactions

**Figure 6:**
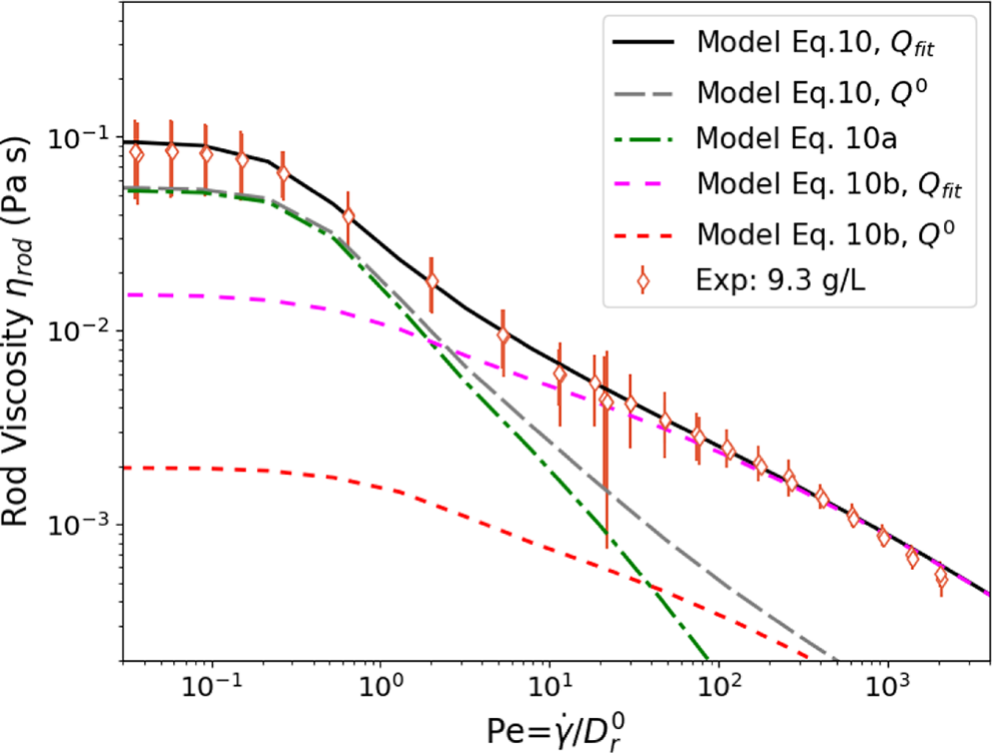
Comparison of the measured rod viscosity (at 9.3 g/L; 0.7 vol %) to the viscosity from the model (eq 10). The contributions from the two terms eqs 10 and 10b are shown as dashed and dotted lines. *Q*^0^ = 0.14 here, and *Q*_fit_ ≈ 1.1.

Figure 6 begins to define the effects of interaction; it illustrates the theoretical terms in eq 10 and how they compare to the data at a single semidilute concentration (9.3 g/L, 0.7 vol %). The viscosity comprises two terms (eqs 10 diffusion, and 10b stresslet); see lines in Figure 6. The previous section began to suggest that interactions between rods contribute significantly to stress, especially at higher concentrations. These effects are more explicit here. At low shear rates, the effect of interactions is mostly described by the change in the effective diffusion coefficient (eqs 8 and 10), consistent with previous work.^14^ At high shear rates, the hydrodynamic stresslet term is larger than the diffusive term, and in contrast to the suggestion that interactions become negligible and dilute behavior is observed at high shear rates,^14^ the data at these previously inaccessibly high shear rates is nearly an order of magnitude greater than the dilute prediction. We see therefore that *Q* must be adjusted (from *Q*^0^ = 0.14 to *Q*_fit_ ≈ 1.1) to account for unexpectedly strong hydrodynamic interactions at a high shear rate. The origin of these interactions is not known, and their possible mechanistic aspects will be discussed below. Note indeed that the form of the stresslet term fits very well the shape of the data, when *Pe* ≳ 10. This motivates the modification of μ_f_ by adjusting factor *Q* in order to characterize the effect of the hydrodynamic interactions.

**Figure 7:**
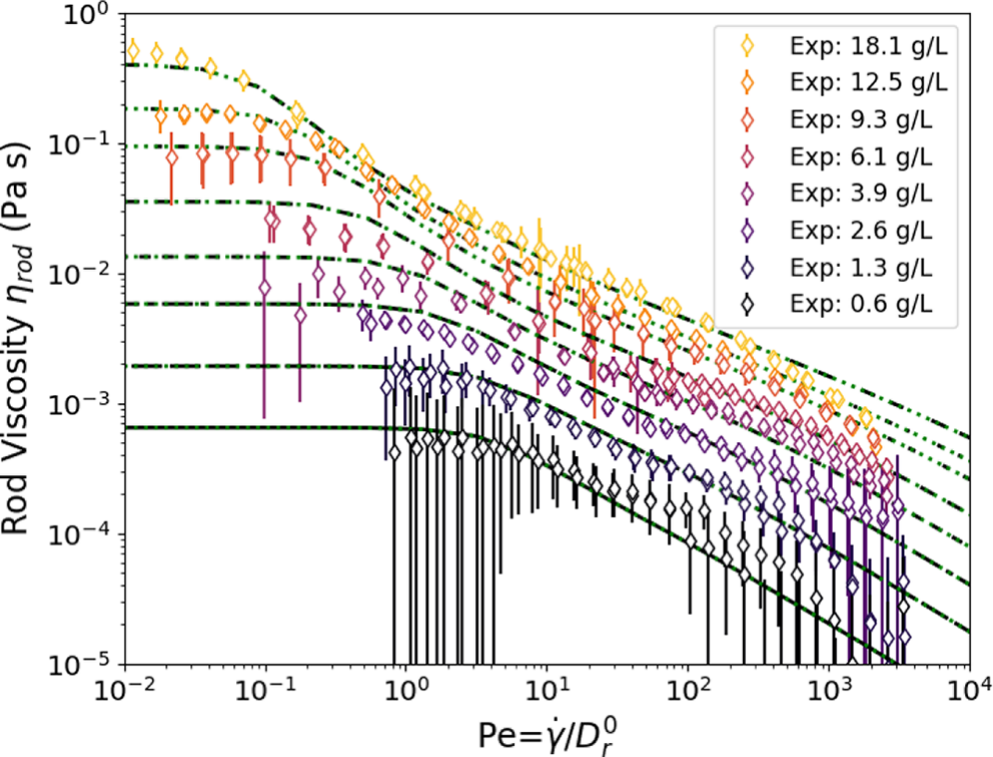
Comparison of the measured rod viscosity to the viscosity from the model (eq 10) including both diffusive interactions and adjusting μ_F_ by changing the effective hydrodynamic interaction term *Q*.

This approach is then applied to all of the semidilute suspension concentrations, fitting the model (eq 10) with a value of *Q* for each concentration (Figure 7), resulting in fairly good agreement with the experiment. While adjusting the magnitude of *Q* does include a fitting parameter and is not predictive, the slope of shear thinning observed in the experiment at high shear rates matches eq 10b well. The results also show reasonably good agreement at low shear rates, and the zero-shear-rate viscosity values compare fairly well with a single value of *c*_1_ = 1350. This shows that the Doi scaling (*nL*^3^)^2^ fairly well describes the measured zero-shear-rate viscosity.

**Figure 8:**
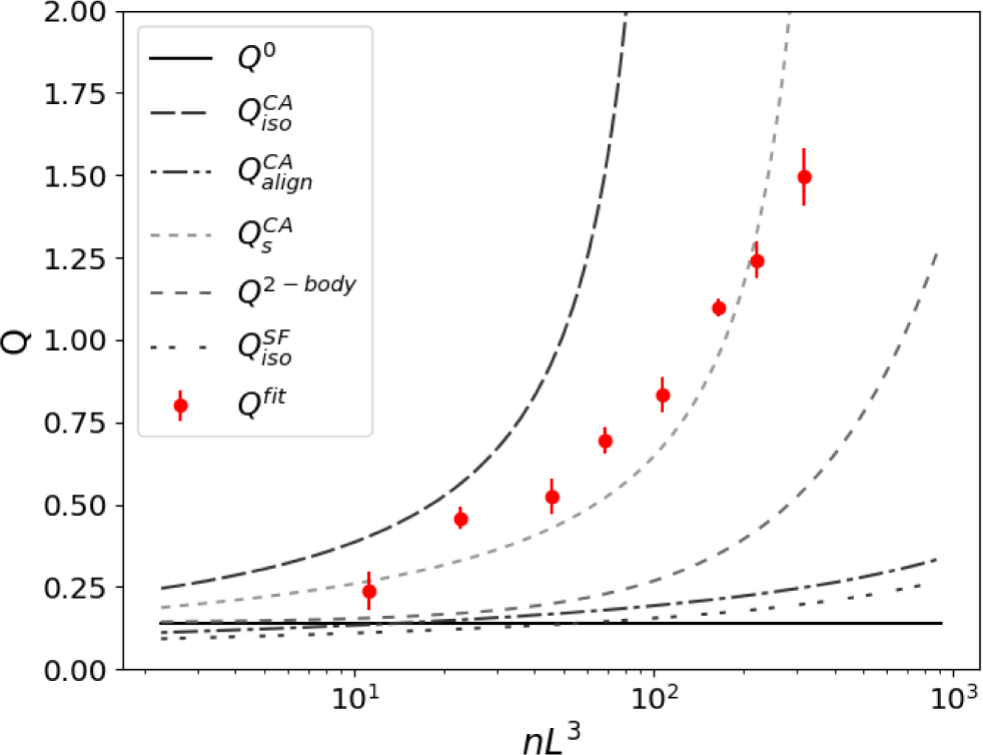
Comparison of different model values of *Q* to fitted values from experiments (symbols) as a function of the concentration. The lines show the dilute limit, *Q*^0^eq 12, two-body theory *Q*^2-body^eq 14, limits from the closest approach for isotropic, , and aligned,  from eq 16 and the isotropic prediction of eq 17. The intermediate value of the closest approach with an *s* = 250 nm is shown in .

The fitted magnitudes of *Q* (see Figure 8) with concentration are compared to different theoretical predictions for the interparticle distance from the literature. In particular, we compare the results of assuming a 2-body interaction, eq 14, a semidilute isotropic configuration, eq 17, and the average closest approach distance, eq 16. Because multiple pairwise interactions occur simultaneously for each rod, a weak dependence of *Q* on concentration is expected from changes in the average interfiber spacing, eq 17, even with an isotropic configuration. The fitted values of *Q* compare most closely with the average closest approach distance assumption and the two-body correction. The expression for the closest approach given in eq 16 with a value of *s* intermediate between the diameter and length, *s* = 250 nm, shows the best agreement with the fitted values. This value of *s* is closer to its isotropic limit and is surprising since at high shear rates where eq 10b is the dominant stress term, the *Pe* number is high and the particles are expected to be highly aligned. The fitted values thus may describe extra stress that arises from the closer hydrodynamic interactions, associated especially with tumbling events that incur local temporary misalignment, which induce other stronger hydrodynamic interactions. One possible mechanism for the increased viscosity is that the rate of tumbling is hindered by the neighboring rods and increases the time in a higher-stress, less aligned, orientation. An interplay of tumbling and hydrodynamic interactions further suggests cascading tumbling of rods; yet, how hydrodynamic interactions influence the frequency of tumbling is yet unknown.

Since the rods are not absolutely stiff, we wondered about the effects of flexibility. We note, however, that a stiffer mutant of the virus exhibits lower intrinsic shear viscosity than the wild-type fd at intermediate shear rates in shear flow (1 to 200) s^–1^ while the stiffer mutant also exhibited lower extensional viscosity than the wild type.^63^ In shear flow, flexibility might increase the tumbling frequency and change the frequency and interaction characteristics, potentially through both hydrodynamic and direct interactions. Buckling may change the tumbling behavior or the effective hydrodynamic volume compared to an isolated rigid rod.^63−66^ These potential consequences are much the same as those exhibited by the minor (slightly faster sedimenting) species detected by AUC (Figure 2), even if the details differ. The transition to buckling behavior can be described by a critical shear rate developed by Chakrabarti et al.^67^ Using the parameters for the fd virus, we estimate the critical shear rate for buckling in dilute conditions to be γ̇_crit_ ≈ 1800 s^–1^, which is *Pe* ≈ 109. At this rate, the viscosity is dominated by the extra hydrodynamic stress fit by an adjustable *Q*, and there is no significant signature of buckling observed in the viscosity at this shear rate (Figure 7).

### Flow-Induced Birefringence

**Figure 9:**
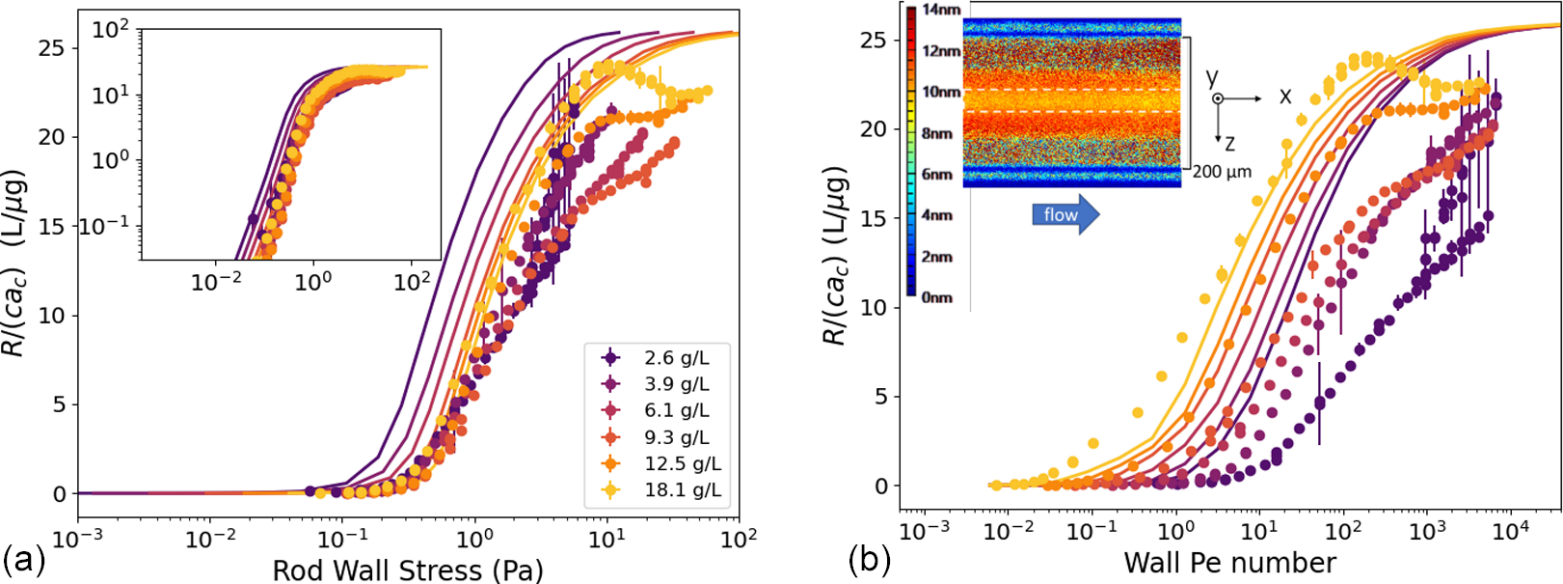
(a) Retardance normalized by capillary diameter and concentration for different concentrations of fd as a function of rod stress at the capillary wall. The solid lines are the mean retardance calculated from the model, as discussed in the text. The error bars indicate the standard deviation in the birefringence over time and in the center of the channel in a region of (512 × 17) superpixels (445 × 14.8 μm). (b) The same data from (a) but plotted as a function of wall *Pe* number. The mean retardance is from the centerline region (marked with a dashed line in the inset raw retardance image).

Next, we compare the retardance measured through the capillary to the model predictions by averaging the contribution to the birefringence from the linear stress gradient across the channel radius; see Birefringence section for details. The raw retardance image is inset in Figure 9b. The mean birefringence is measured from the center region of that (and each) image, where the retardance is at a local minimum. For small ±z from the capillary center, the measured total retardance increases parabolically because the mean and minimal stresses are greater through those optical paths that do not pass through the center of the capillary (where the stress is zero). Various other factors influence the retardance image, especially further from the capillary center, where the curvature of the cylindrical capillary refracts the optical path. The measured mean birefringence per concentration is shown as a function of rod stress at the capillary wall in Figure 9 and compared to the model predictions. The model stress using the fitting parameters for the high shear rate, shown in Figure 7, is used to determine the rod stress from the applied pressure drop across the capillary.

The highest measured retardance value is matched to the 18.1 g/L system to determine a saturated birefringence value of Δ*n*_sat_/c = 2.6 × 10^–5^ L g^–1^. This value is slightly less than previous measurements of nematic systems of Δ*n*_sat_/*c* = 3.8 × 10^–5^ L g^–1^ measured by Purdy et al.^51^ This may be an indication that a fully aligned state has not been reached at the highest shear rates. Using a literature value of 3.8 × 10^–5^ L g^–1^, a maximum order parameter of approximately 0.7 is reached in our experiments, although measurement uncertainties and differences in measurement methods make an accurate comparison difficult. Indeed, another much lower value of Δ*n*_sat_/*c* = 1.3 × 10^–5^ L g^–1^ was recently reported.^68^ Furthermore, that recent study compared alignment in extensional and shear flows, investigating the effect of flow kinetics on alignment, and showed that even in strong shear flows, fd virus did not reach a highly aligned state compared to extensional flows.^68^

The data show a qualitatively similar behavior to the model as a function of rod stress (shown as lines in Figure 9a) at low values of mean birefringence. The mean birefringence initially increases with a power law of 2 before saturating at a constant; see the inset in Figure 9. The mean retardance calculated from the model predicts a slightly higher stress for the onset of alignment as concentration increases. The largest discrepancy between the model and experiment is for the low concentration data, which is more susceptible to measurement uncertainties. This is even more evident when data are plotted as a function of wall *Pe* number in Figure 9b. The lower concentrations show a slower rate of increase in birefringence and do not reach a fully saturated value over the range of accessible stresses.

At higher values of mean birefringence, the high concentration shows a peak and then decreases as stress reaches its highest value. We note that at the high shear rates, there is a significant contribution of the solvent stress to the total stress for the lower concentrations, and consequently, there is greater uncertainty of the rod stress at lower concentrations.

The decrease in viscosity occurs even as the rods reach a nearly fully aligned orientation at high shear rates. This behavior is known since Hinch and Leal’s calculations,^31^ which demonstrated that viscosity depends on even slight deviations from perfect alignment, showing that orientational order and shear effects change nonlinearly.

The overshoot in alignment for the high concentrations is unexpected, considering that theory predicts a monotonic increase in alignment with stress. Curiously, the peak in the alignment (at 18.1 g/L) and its shoulder (at 12.5 g/L) coincide with the predicted dilute buckling transition (Figure 9). It is not clear, however, what this peak signifies about the rod dynamics, either their tumbling or buckling, since there is not a corresponding feature in viscosity (Figure 7). We suppose that the overshoot is related to particle interactions while tumbling, in particular the occasional tumbling at high shear rates. At high concentrations, there may be a competition between interactions that decrease tumbling and interactions that increase tumbling. At intermediate shear rates, there may be more interactions that increase alignment, while at even higher rates, coupling of tumbling dynamics between rods might decrease alignment. There is precedent of an overshoot in alignment; it was observed recently in flow-birefringence studies of a more flexible rod Pf1.^69^ The mechanism responsible for this feature, however, has not yet been discovered.

### Orientation Relaxation

**Figure 10:**
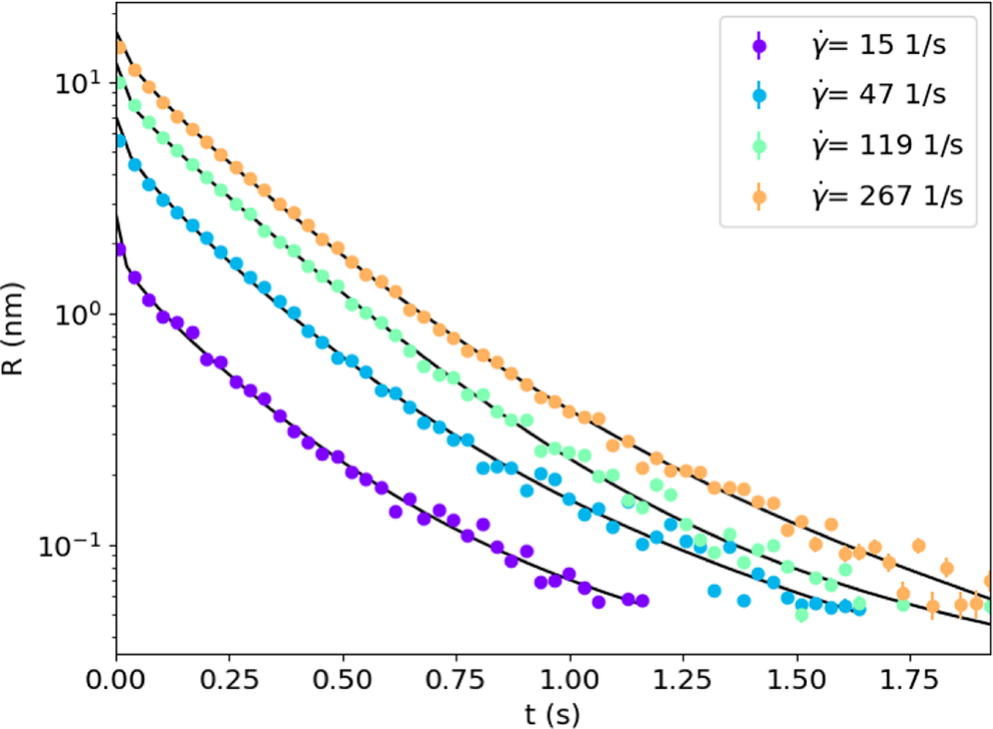
Relaxation of retardance after flow cessation for *c* = 12.5 g/L at different values of the initial shear rate. The data are fit with a sum of exponential functions (see the text and Figure 11).

The relaxation behavior of the rod orientation is investigated by stopping the pressure-driven flow with a pneumatic valve. This stops the flow within approximately 20 ms, and a decrease in the mean retardance is recorded as a function of time (Figure 10). While a single exponential relaxation is expected if there are no rod interactions, a more complex relaxation process occurs as interactions change the effective diffusion coefficient with orientation (see eq 8). At early times, the relaxation slows as it progresses toward an isotropic configuration, while the effective diffusivity decreases with time, as the interactions between rods grow stronger. At long times, the relaxation follows a single exponential relaxation with a time scale related to the effective diffusion coefficient in an isotropic orientation. We aim to compare the measured terminal relaxation to the time scale expected from theory. To do this, we compare the relaxation as a sum of an exponential function to represent the terminal relaxation and a stretched exponential function to represent the early time behavior: *R* = *R*(*t* = 0) (*f* exp((−*t*/τ_1_) + (1 – *f*) exp((−*t*/τ_2_)^β^). The retardance at flow cessation is taken as *R*(*t* = 0). Examples of this function fit to experimental data from the *c* = 12.5 g/L system are shown in Figure 10.

The short-time behavior changes with concentration and deviates more significantly from a monoexponential response at higher concentrations. For a given concentration, there are only slight deviations in the rate of this stretched exponential behavior, as seen in Figure 10, indicating that the degree of initial alignment does not significantly change the overall relaxation dynamics. In the model, the stretching exponent changes slightly as the shear rate increases due to the broadening of the change in the relaxation time scales from a highly aligned state.

**Figure 11:**
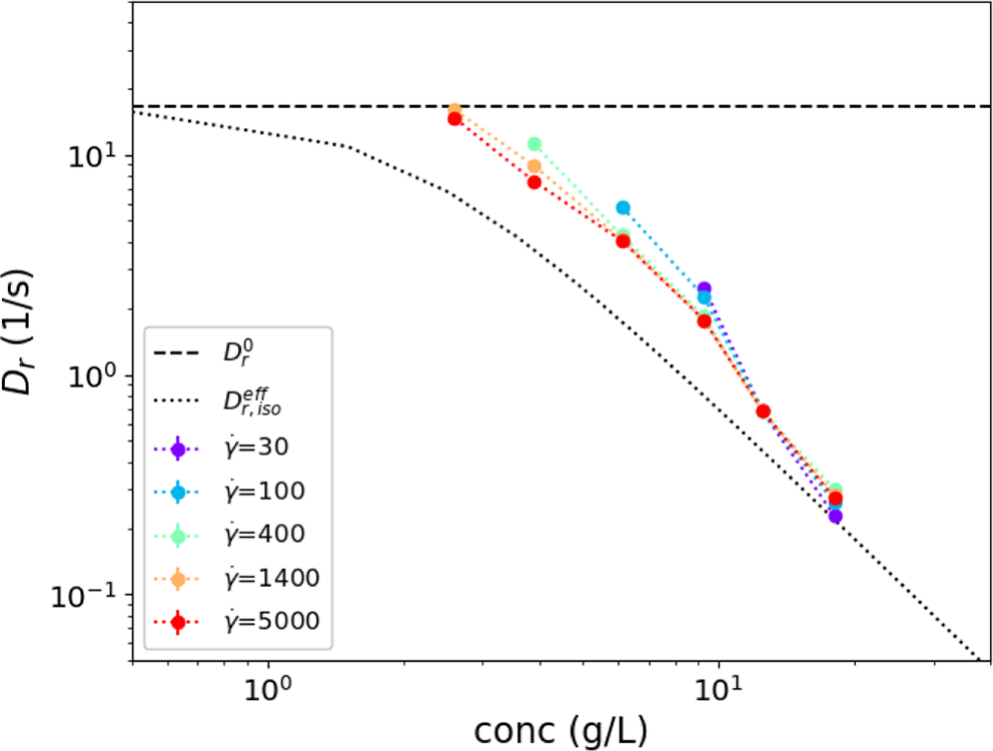
Measured diffusion coefficient from terminal relaxation *D*_r_ = 1/(6τ_1_) for relaxation of retardance after flow cessation for different values of initial shear rate and concentration. The data are compared to model predictions, which are independent of the shear rate for the model. The effective diffusion coefficient for an isotropic configuration, , is shown as as a dotted line.

We compare the fitted terminal time scale, τ_1_, as a diffusion coefficient by *D*_r_ = 1/(6τ_1_) for both the experiment and model in Figure 11. Experimental values at low concentrations and low shear rates are excluded due to insufficient signal to measure the relaxation. The model relaxation is calculated from the average birefringence starting from an initial steady-shear condition and then setting the applied shear rate to zero at *t* = 0. The model values for τ_1_ are independent of shear rate, and the corresponding diffusion coefficient from the model is slightly lower than the effective diffusion coefficient in eq 7, because of the concentration (Φ) dependence that appears in a term that hinders the relaxation back to isotropic orientation. Like these modeled diffusivities, the measured diffusion coefficient varies negligibly with shear rate and decreases similarly with concentration. Furthermore, the experiments show a slightly higher value of  compared with the predicted values.

## Conclusions and Perspective

In this work, we have used rheology and flow-induced birefringence to study the flow behavior and microstructure of semidilute rod-like fd-virus suspensions at high shear rates. No changes in the material, such as stress-induced damage, are apparent. The viscosity shows the expected scaling at low shear rates resulting from hindered diffusion in semidilute concentrations, while the viscosity measured at high shear rates indicates surprisingly strong hydrodynamic interactions. By fitting the viscosity coefficient, these interactions appear comparable to those hydrodynamic interactions expected from a nearly isotropic structure dominated by the closest approach. These strong hydrodynamic interactions are likely associated with hindered rod tumbling and therefore suggest cascading tumbling of the rods.

Although it has been shown previously that interactions between rods are reduced by alignment,^17^ significant interactions remain here when aligned at high shear rates. From the measurement of viscosity, we infer that these interactions continue to have a semidilute dependence on concentration. At low semidilute concentrations, they are most consistent with the distance of closest approach near that of an isotropic configuration (Figure 8), which suggests that, while the rods are aligned, they spend a fraction of their time tumbling and the misorientation away from the shear plane during tumbling is an important part of their interaction. At higher semidilute concentrations, the effect of interactions is intermediate between this multibody and a two-body approximation (Figure 8). It is not clear whether an increase in dynamical states such as tumbling would change the shear thinning exponent, but the good comparison with eq 10b seems to indicate that the stress still follows the same shear thinning behavior.

The molecular orientation determined from flow birefringence is qualitatively similar to model predictions of the alignment. However, while the alignment is expected to grow monotonically with stress, the highest-concentration suspension strikingly exhibits a peak in alignment at intermediate to high stress. At high concentration, there may be a competition between excluded volume interactions that act to decrease tumbling at intermediate shear rates, but at higher rates, the interactions lead to increased tumbling and misalignment. The lower concentrations do not exhibit this peak, yet their alignment is also somewhat lower than expected from theory. Perhaps this weaker alignment and the stronger viscosity under these high-shear conditions arise from the same mechanism. Hydrodynamic interactions may trigger more frequent molecular tumbling away from the shear plane, which further increases hydrodynamic interactions and reduces alignment. The flexibility of the rod-like particle is also likely to affect the tumbling frequency and change the characteristics of interactions caused by tumbling.

A number of parameters influence the shear dynamics of rodlike suspensions, from distributions of filament length and aspect ratios, to persistence length and volume fraction.^17,70,71^ The effects of flexibility and perhaps buckling are not determined here but could be analyzed on these samples prepared in D_2_O using small-angle neutron scattering. The large aspect ratio of the fd virus suspensions results in a relatively large viscosity increase per volume of rods and may display a more significant increase in viscosity at high shear rates compared to shorter rods. High shear rate measurements on stiffer rod suspensions with smaller aspect ratios would provide insight into how universal this increased viscosity is at high shear rates. Hydrodynamic interactions between rods in extensional flows might also distinguish the effect of tumbling.

## Footnotes

a Certain commercial equipment, instruments, software, or materials, commercial or non-commercial, are identified in this paper in order to specify the procedure adequately. Such identification is not intended to imply recommendation or endorsement of any product or service by the National Institute of Standards and Technology, nor is it intended to imply that the materials or equipment identified are necessarily the best available for the purpose.
